# Synthesis, Photophysical Characterization, and Photoinduced Antibacterial Activity of Methylene Blue-loaded Amino- and Mannose-Targeted Mesoporous Silica Nanoparticles

**DOI:** 10.3390/molecules20046284

**Published:** 2015-04-09

**Authors:** Oriol Planas, Roger Bresolí-Obach, Jaume Nos, Thibault Gallavardin, Rubén Ruiz-González, Montserrat Agut, Santi Nonell

**Affiliations:** Institut Químic de Sarrià, Universitat Ramon Llull, Via Augusta 390, 08017 Barcelona, Spain; E-Mails: oriol.planas@iqs.url.edu (O.P.); rogerbresolio@iqs.url.edu (R.B.-O.); jaimenosa@iqs.url.edu (J.N.); thibault.gallavardin@gmail.com (T.G.); ruben.ruiz@iqs.url.edu (R.R.-G.); montserrat.agut@iqs.url.edu (M.A.)

**Keywords:** antimicrobial photodynamic therapy (aPDT), singlet oxygen (^1^O_2_), mesoporous silica nanoparticle (MSNP), methylene blue, drug delivery system, mannose, *E. coli*, *P. aeruginosa*

## Abstract

Over the last 20 years, the number of pathogenic multi-resistant microorganisms has grown steadily, which has stimulated the search for new strategies to combat antimicrobial resistance. Antimicrobial photodynamic therapy (aPDT), also called photodynamic inactivation, is emerging as a promising alternative to treatments based on conventional antibiotics. We have explored the effectiveness of methylene blue-loaded targeted mesoporous silica nanoparticles (MSNP) in the photodynamic inactivation of two Gram negative bacteria, namely *Escherichia coli* and *Pseudomonas aeruginosa*. For *E. coli*, nanoparticle association clearly reduced the dark toxicity of MB while preserving its photoinactivation activity. For *P. aeruginosa,* a remarkable difference was observed between amino- and mannose-decorated nanoparticles. The details of singlet oxygen production in the nanoparticles have been characterized, revealing the presence of two populations of this cytotoxic species. Strong quenching of singlet oxygen within the nanoparticles is observed.

## 1. Introduction

Over the last 20 years, pathogenic microorganisms have become a global threat as the third most significant cause of death in Europe and the second worldwide, with 17 million annual deaths [1]. This rise in the malignancy of pathogenic infections is attributed to the emergence of new infectious diseases as well as to the re-emergence of diseases previously controlled, owing to the combined effect of factors such as excessive or inappropriate prescription of antibiotics, the failure of some patients to complete their treatment regime, and the expansion of poverty areas where prophylactic measures are lacking [2]. The most notorious drug-resistant pathogens are generally reported as “ESKAPE,” standing for *Enterococcous faecium*, *Staphylococcus aureus*, *Klebsiella pneumonia*, *Acinetobacter baumanii*, *Pseudomonas aeruginosa*, and *Escherichia coli* [3].

Not only has the number of pathogenic multi-resistant microorganisms steadily increased but also the approval of new antibiotics has dramatically slowed down, posing a severe problem to global health [3]. Antimicrobial photodynamic therapy (aPDT), also called photodynamic inactivation (PDI), is currently being actively explored as an alternative to conventional antimicrobial treatments based on antibiotics. aPDT is based on the application of a photoactive drug, referred to as the photosensitizer (PS), that is irradiated with *per se* harmless visible light. As a consequence of light absorption, the photoexcited PS forms reactive oxygen species (ROS), such as singlet oxygen (^1^O_2_) or superoxide radicals, which oxidize essential biological substrates in the vicinity of the PS, leading to cell death [4]. In view of the variety of molecular targets and the ability to inflict damage to a pathogen even without internalization of the PS, aPDT holds great potential for the inactivation of bacteria with little risk of developing resistance. In fact, selection of aPDT-induced resistant pathogens *in vitro* [5,6,7] or *in vivo* [8] has not been reported so far. 

**Scheme 1:**
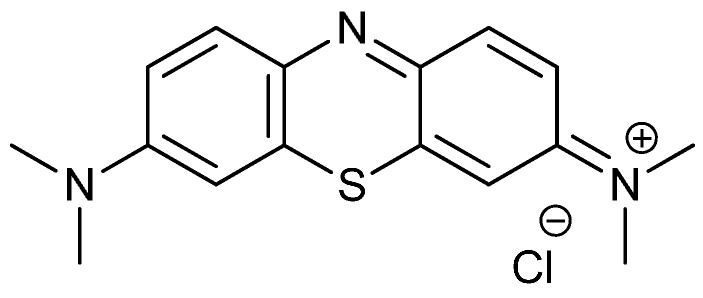
Chemical structure for MB.

Many different PSs have been tested as aPDT agents over the last few years. The most effective ones invariably bear positive charges on their structure at physiological pH. This is the case for, e.g., phenothiazines [9,10] like methylene blue (MB) (see Scheme 1), porphyrins [11], phthalocyanines [12], and porphycenes [13]. Their high activity against microbial cells, including hard-to-kill Gram-negative bacteria, is due to the negative net charge of their cell wall, which facilitates binding of the PS through electrostatic interactions [14,15]. On the other hand, this effect is so general that aPDT shows little selectivity towards pathogenic microorganisms. 

Recent advances in nanotechnology offer an opportunity to overcome the limitations of traditional PSs in aPDT. Nanoparticles can be used as drug delivery systems for the PS and may confer enhanced selectivity by grafting targeting ligands onto their surface for selective recognition by receptors on the pathogenic cell wall. Among the nanovehicles used in nanomedicine [16], mesoporous silica nanoparticles (MSNP) are of great interest in targeted PDT owing to their biocompatibility, high PS loading capacity, and ease of surface functionalization [17,18]. 

Although huge efforts have been made to spread the use of MSNP for the treatment of several diseases, particularly cancer [19,20,21], only a few examples have been reported so far describing their application to bacterial infections. We therefore set out to investigate the effectiveness of MB-encapsulated targeted MSNPs in the inactivation of two ESKAPE Gram negative bacteria. Specifically, we have decorated MSNP with two different targeting motifs, amino groups (AMSNP) and mannose sugars (MMSNP) [22], loaded them with MB, and evaluated their photophysical properties and photodynamic activity against *Escherichia coli* and *Pseudomonas aeruginosa*.

## 2. Results and Discussion

### 2.1. Synthesis, Functionalization, and Loading of MB onto Mesoporous Silica Nanoparticles

**Scheme 2:**
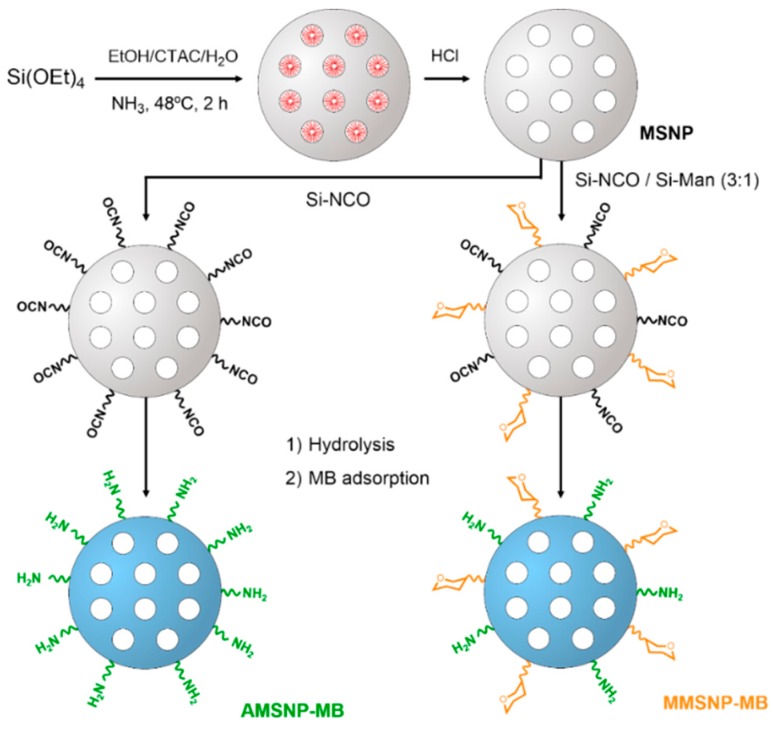
Synthesis of MSNP, AMSNP-MB, and MMSNP-MB.

The procedure for the synthesis of mesoporous silica nanoparticles (MSNPs), as well as their functionalization with amino- and mannose-moieties, is illustrated in Scheme 2. MSNPs were prepared via the sol-gel process under high dilution conditions. After the removal of the CTAC surfactant template using HCl, different aliquots of MSNP were treated with 3-(triethoxysilyl)propyl isocyanate (Si-NCO) or a mixture of 3-(triethoxysilyl)propyl isocyanate and *N*-(*d*-mannose)-*N*’-(3-(triethoxysilyl)propyl))-urea in 3:1 molar ratio (Si-NCO/Si-Man). Subsequent hydrolysis of the isocyanate groups in water followed by loading with MB through electrostatic interactions yielded the corresponding MB-loaded amino- or mannose-modified mesoporous silica nanoparticles (AMSNP-MB and MMSNP-MB, respectively). 

The modified MSNPs were characterized by their size and ζ-potential as well as by infrared (IR) spectroscopy. Dynamic light scattering (DLS) shows that the starting MSNPs had a hydrodynamic diameter of about 160 nm and a ζ-potential of −40 mV. Upon addition of MB, followed by functionalization with the silanes and subsequent hydrolysis of the unreacted NCO groups, the diameter of the nanoparticles increased up to 200 nm and the ζ-potential became less negative (Table 1). 

**Table 1:** Size, ζ-potential, and MB-loading ratio (MB-LR) of the mesoporous nanostructures.

	Size/nm	ζ-potential/mV	MB-LR (%)
MSNP	160	−40	
AMSNP-MB	200	−25	73
MMSNP-MB	180	−20	94

**Figure 1:**
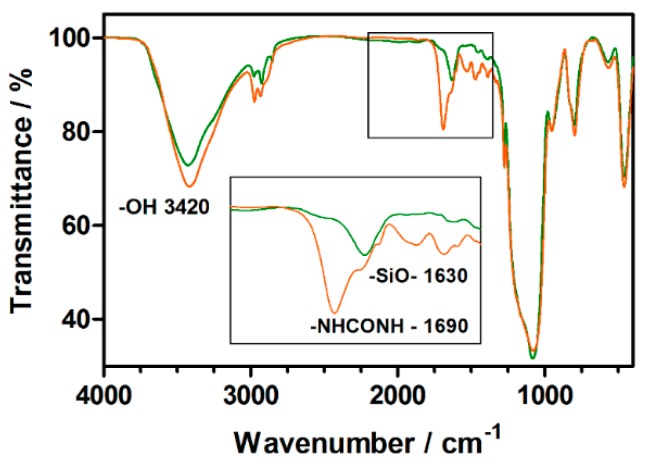
IR spectra of AMSNP (green) and MMSNP (orange).

Moreover, a comparative study of the IR-spectra of AMSNP and MMSNP (Figure 1) confirmed the efficient functionalization of MMSNP with mannose, as demonstrated by the presence of a band centered at 1690 cm^−1^, characteristic of urea bridges, in the spectrum of MMSNP but not in that of AMSNP. On the other side, the –NCO stretching band is not observed in any of the samples, ruling out an ineffective hydrolysis of these groups. Finally, the dominant –O-H stretching band centered at 3420 cm^−1^ precludes the observation of the –N-H absorption.

### 2.2. Photophysical Properties of MB-Loaded Nanoparticles

#### 2.2.1. Absorption and Fluorescence Spectra

**Figure 2:**
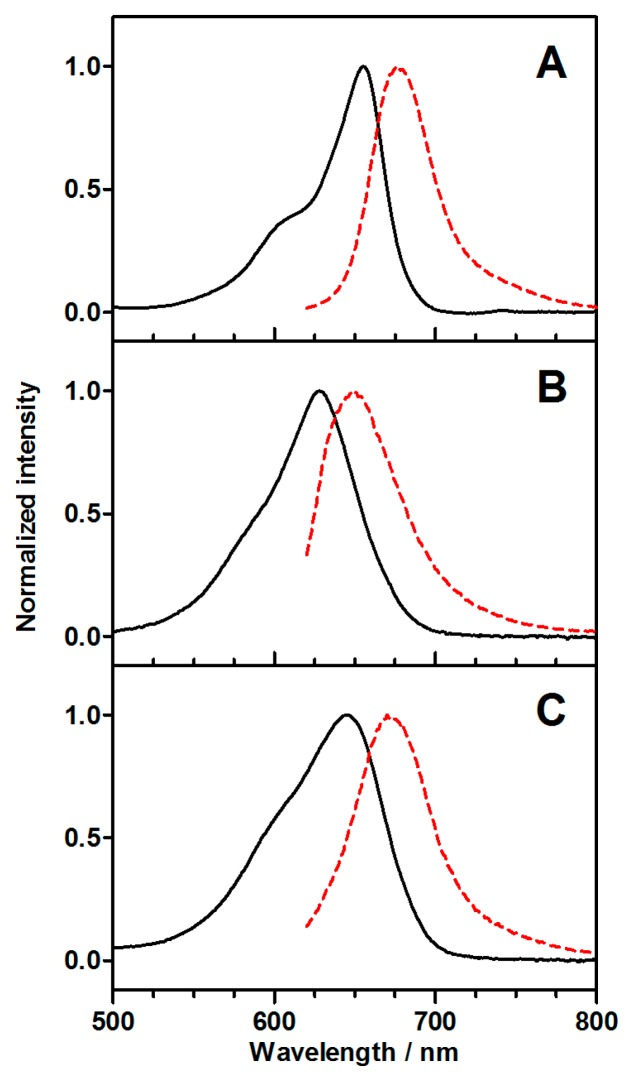
Absorption (black solid line) and emission (red dashed line) spectra of MB (**A**), AMSNP-MB (**B**), and MMSNP-MB (**C**) in EtOH upon excitation at 600 nm.

Figure 2 shows the visible absorption and fluorescence spectra of MB, AMSNP-MB, and MMSNP-MB in EtOH. The spectra of AMSNP-MB and MMSNP-MB are blue-shifted relative to those of free MB. This indicates that all MB is adsorbed on the surface of the nanoparticles, where it experiences a different microenvironment. Consistent with this, the relative intensity between the shoulder and the maximum of the absorption bands also varies from compound to compound, leading to less-structured bands for AMSNP-MB and MMSNP-MB. 

#### 2.2.2. Fluorescence Kinetics

Time-resolved fluorescence decays were recorded at the maximum of the emission bands, as described in the Experimental section. While the decay was monoexponential for free MB, biexponential decays were observed for MB adsorbed onto the nanoparticles, which suggests two different populations of the PS. The relative amplitudes of the two components are given in Table 2 and it is noteworthy that the dominant emission is the one with the longest lifetime, roughly twice as long as that observed for free MB. 

**Table 2:** Decay kinetics and relative intensity of all tested nanoparticles in ethanol.

	Component	τ_F_/ns	Amplitude (%)
MB	1	0.8	100
	2	---	---
AMSNP-MB	1	0.8	33
	2	1.5	67
MMSNP-MB	1	0.7	13
	2	1.4	87

#### 2.2.3. Near-Infrared Phosphorescence Decays

**Figure 3:**
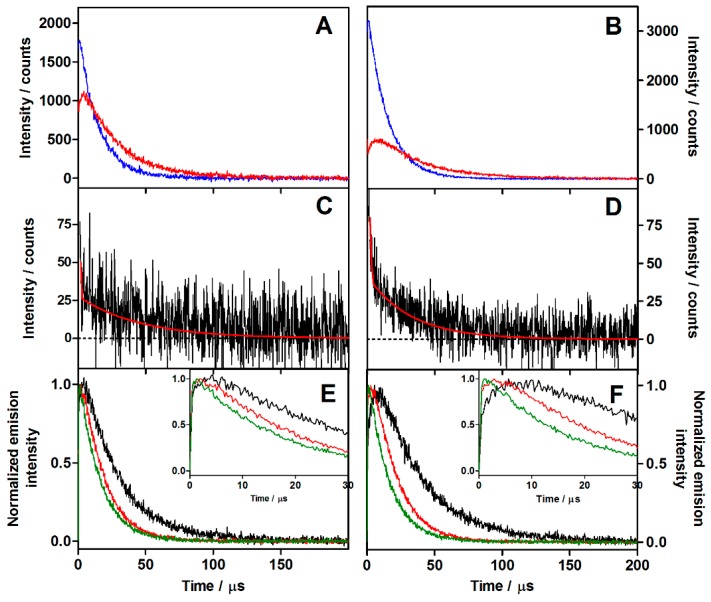
(**A**) Time-resolved ^1^O_2_ phosphorescence (λ_obs_ = 1270 nm) of optically matched solutions of MB (blue) and AMSNP-MB (red) excited at 532 nm. (**B**) Time-resolved ^1^O_2_ phosphorescence of optically matched solutions of MB (blue) and MMSNP-MB (red) at 532 nm. (**C**) Time-resolved phosphorescence emission at 1110 nm of AMSNP-MB. (**D**) Time-resolved phosphorescence emission at 1110 nm of MMSNP-MB. (**E**) Normalized time-resolved ^1^O_2_ phosphorescence of AMSNP-MB in air, (black) oxygen saturated (red) and in 100 mM acetic acid aerated solution (green). (**F**) Normalized time-resolved ^1^O_2_ phosphorescence of MMSNP-MB in air (black), oxygen saturated (red), and in 100 mM acetic acid aerated solution (green).

Pulsed-laser irradiation of MB, AMSNP-MB, and MMSNP-MB suspended in ethanol produced clear time-resolved ^1^O_2_ phosphorescence signals at 1270 nm (Figure 3). The ^1^O_2_ signal for MB grew with a lifetime of 0.2 μs and decayed monoexponentially with a time constant of 15 μs. An additional rise component was needed to fit the data for MB associated to the MSNPs. Specifically, the ^1^O_2_ signal for AMSNP-MB was found to show biphasic growth kinetics with time constants of 0.2 μs and 3.3 μs, respectively, and decayed monoexponentially with a time constant of 26 μs (Figure 3A). The lifetimes were slightly longer for MMSNP-MB: 0.2 μs, 4.6 μs, and 34 μs, respectively (Figure 3B). 

In order to assign the observed lifetimes, additional emission decays were recorded at 1110 nm, where the phosphorescence from MB’s triplet state can be monitored independently. The signal was found to decay biexponentially with time constants 0.2 μs and 24 μs for AMSNP-MB, and 0.2 μs and 35 μs for MMSNP-MB (Figure 3C,D), which match two of the three components observed in the ^1^O_2_ phosphorescence signals at 1270 nm. This is consistent with two MB populations, as observed in the fluorescence experiments. Moreover, the very different lifetimes indicate very different exposure to oxygen for the two triplets. This was confirmed by saturation of the suspensions with oxygen, which completely eliminated the shortest component and reduced the lifetime of the longest one to 16 μs for AMSNP-MB and 18 μs for MMSNP-MB (Figure 3E,F). 

Returning to the ^1^O_2_ phosphorescence signals at 1270 nm, it must be concluded that the third component (3.3 μs for AMSNP-MB and 4.6 μs for MMSNP-MB) corresponds to the decay of ^1^O_2_ in each suspension. This lifetime is much shorter than that observed for free MB in EtOH (15 μs), therefore it must be concluded that ^1^O_2_ is quenched by the nanoparticles. This was confirmed by addition of 100 mM acetic acid, which protonated the amino groups of the nanoparticle surface, rendering them cationic with the concomitant release of MB due to electrostatic repulsion. Under such conditions, the ^1^O_2_ phosphorescence signals showed the same kinetics as those for free MB in ethanol (Figure 3E,F).

### 2.3. Photodynamic Inactivation of Gram-Negative Bacteria

**Figure 4:**
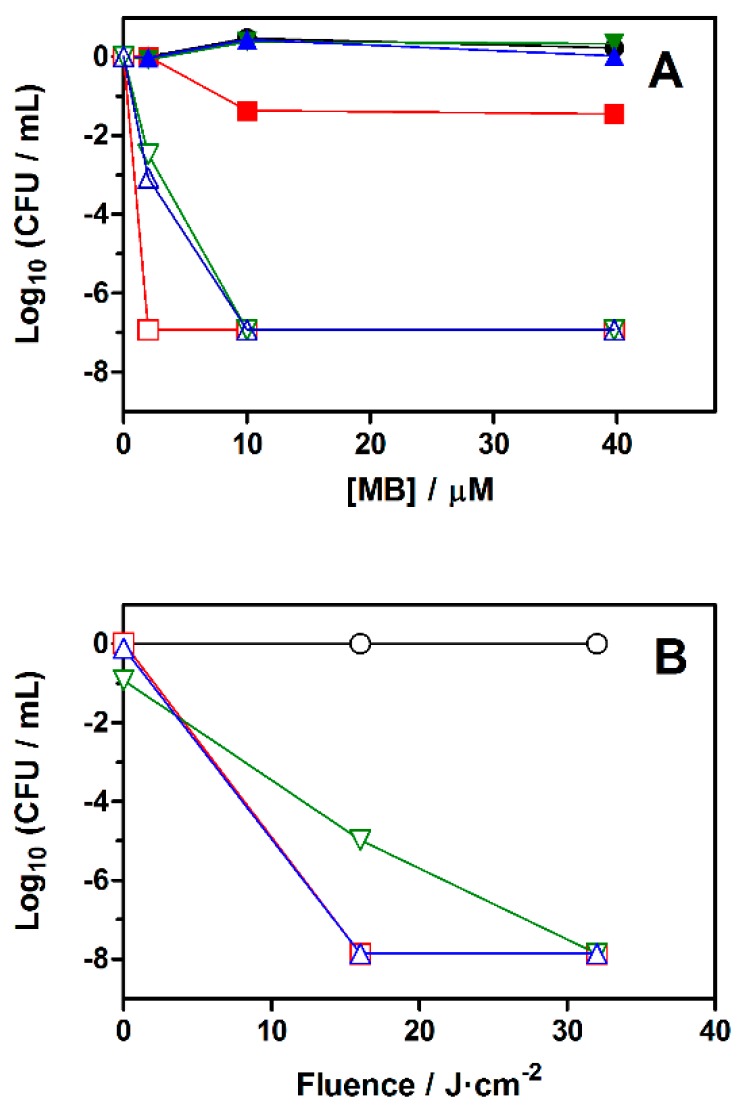
(**A**) Survival curves of *E. coli* incubated with different concentrations of MB in the dark (closed symbols) and after being exposed to a light fluence of 16 J/cm^−2^ at 652 nm (open symbols). (**B**) Survival curves of *P. aeruginosa* incubated with 10 μM MB after receiving increasing light fluences at 652 nm. Control experiments and cells incubated with free MB, AMSNP-MB, and MMSNP-MB are represented by circles, squares, inverted triangles, and triangles, respectively.

In order to assess the antibacterial potential of the nanostructures, a series of microbiological assays were conducted on *E. coli* and *P. aeruginosa* suspensions. In the absence of light, MB, incubated for 30 min at 2 μM concentration, induced no dark toxicity to *E. coli* irrespective of the vehicle used for delivery. However, when the MB concentration was increased to 10 μM, and even more so at 40 μM, it was observed that free MB reduced the survival fraction by almost 2-log_10_, whereas MB bound to the nanosystems was still devoid of any measurable dark toxicity (Figure 4A). This is in line with previous results for other drug delivery systems [23,24]. Irradiation of the bacteria pre-incubated with MB with a 16 J/cm^2^ fluence of red light reduced their survival fraction in a concentration-dependent manner, MB in free form being more phototoxic than when associated to the nanoparticles. There was no appreciable difference between the two types of nanoparticles (Figure 4A).

Regarding *P. aeruginosa*, no dark toxicity differences were detected among the three vehicles at 10 μM MB (Figure 4B). However, while both MB and MMSNP-MB reduced 8-log_10_ the bacterial survival fraction upon exposure 16 J/cm^2^, only a 5-log_10_ reduction was observed for AMSNP-MB. For this nanostructure, higher light fluences, up to 32 J/cm^−2^, were needed to completely inactivate *P. aeruginosa*. 

### 2.4. Discussion 

The absorption and fluorescence spectra of AMSNP-MB and MMSNP-MB are blue-shifted with respect to free MB. This indicates that MB is confined inside the MSNP, as the shift is dependent on the microenvironment polarity. In addition, the absorption spectra of both MSNP-MBs are less structured than the equivalent for free MB, pointing to a partial MB dimerization inside the MSNPs [25].

Regarding the time course of the fluorescence, both nanoparticles show biexponential decays, which suggest two different populations of MB. We ascribe them to MB molecules adsorbed on the outer surface of the nanoparticles and on the walls of the inner mesopores, respectively. This is consistent with the observed lifetimes (close to that of free MB for the externally-bound, solvent-exposed MB molecules and longer for those in the mesopores) as well as with their relative amplitudes (larger for those in the mesopores, in agreement with the higher contribution of the mesopore walls to the total nanoparticle surface). It is remarkable that mesopore localization is favored even more in MMSNP-MB as compared to AMSNP-MB (see Table 2).

The two-population interpretation is also consistent with the MB and ^1^O_2_ phosphorescence results. Thus, two triplet MB phosphorescence lifetimes were observed in the MSNP-MB nanosystems: the shortest value of 0.2 μs is close to that of free MB and is therefore assigned to MB molecules on the external surface of the nanoparticles, while the longest values (26–34 μs depending on the type of nanoparticle) are assigned to MB molecules in the mesopores, where they are more shielded from oxygen.

In line with this view, two populations of ^1^O_2_ are considered in the analysis of the ^1^O_2_ phosphorescence data. The ^1^O_2_ luminescence signal can be described by Equation (1):

(1) $$S\left(t\right)=S_{01}\times \frac{{\text{τ}}_{\Delta }}{{\text{τ}}_{\Delta }-{\text{τ}}_{\text{T}1}}\times (e^{-t/\tau_{\Delta }}-e^{-t/\tau_{\text{T}1}})+S_{02}\times \frac{\tau_{\Delta }}{\tau_{\Delta }-\tau_{\text{T}2}}\times (e^{-t/\tau_{\Delta }}-e^{-t/\tau_{\text{T}2}})$$

where *S*(*t*) is the ^1^O_2_ signal intensity at time *t*, *S*_01_ and *S*_02_ are the contributions of each ^1^O_2_ population to the observed signal, τ_∆_ is the ^1^O_2_ lifetime, and τ_T1_ and τ_T2_ are the triplet-state lifetimes [26]. This model has recently been used by Torra *et al.* to analyze the ^1^O_2_ luminescence of the flavin-binding protein PP2 L30M, which analogously shows two ^1^O_2_ populations [27].

**Figure 5:**
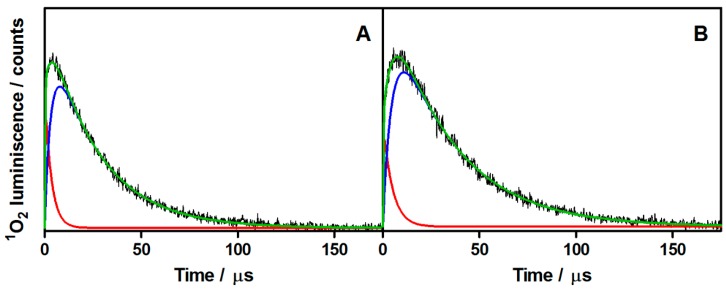
Time-resolved ^1^O_2_ phosphorescence for AMSNP-MB and MMSNP-MB (**A,B**, respectively) in air-saturated absolute ethanol dispersion. Fitted function in green. Contributions of triplet 1 and 2 in red and blue, respectively. λ_exc_ = 532 nm; λ_obs_ = 1270 nm.

Figure 5 shows the plot of the experimental ^1^O_2_ luminescence (black) and its fitting (green) according to Equation 1. The contributions of each population are plotted individually (red for S_01_ and blue for S_02_) for AMSNP-MB and MMSNP-MB (A and B respectively). 

Comparison of the two individual components shows that the ^1^O_2_ signal arises mainly from the longer-lived triplet in the nanoparticle’s mesopores, where oxygen diffusion is more difficult, while the contribution from MB molecules bound to the outer surface of the nanoparticle is comparatively less important (less than 10%).

The ^1^O_2_ lifetime in the MSNPs suspensions (3.3 μs for AMSNP-MB and 4.6 μs for MMSNP-MB) is substantially shorter than in ethanol (15.5 ± 3.5 μs) [28,29]. Three contributions may account for this observation: (i) quenching by free amino groups on the nanoparticle surface, [30,31], (ii) quenching by hydrogen-bonded water and silanol groups in the mesopores [32,33], and (iii) enhancement of these processes by the increased wall collision frequency in the narrow silica mesoporous channels [32].

**Figure 6:**
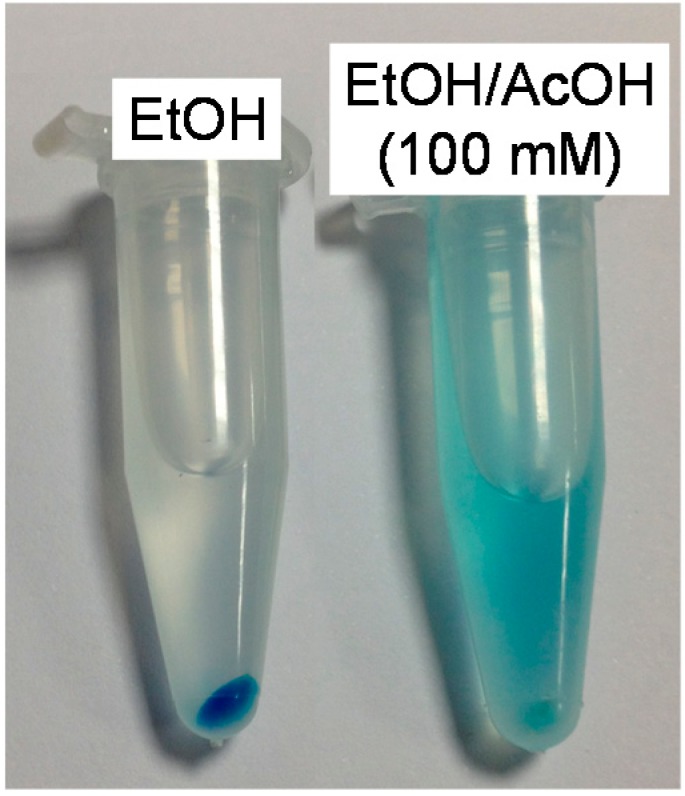
One milliliter of AMSNP-MB in EtOH (left) and in mixture EtOH/AcOH (100 mM, right) centrifuged for 10 min at 10000 rpm. A blue pellet and a colorless supernatant are observed for AMSNP-MB in EtOH, whereas a blue solution and a faint blue pellet are devised in 100 mM AcOH.

It is also interesting to note that in acidic ethanolic media (100 mM acetic acid) the amino groups of the MSNP are protonated and thus positively charged. Electrostatic repulsion causes the positively-charged MB molecules to be released from the nanoparticle to the medium, as illustrated in Figure 6. The kinetics of ^1^O_2_ under these conditions is, understandably, essentially the same as for free MB.

Photodynamic inactivation assays of *E. coli* and *P. aeruginosa* reveal that the antibacterial activity of MB is not affected substantially when it is delivered either using MSNPs or in free form, whereas, in *E. coli*, its dark toxicity is decreased by incorporation to the MSNPs. There is no clear dependence on the microorganism, as observed also when some antibiotics or disinfectants are used. In the case of *P. aeruginosa*, a remarkable difference is observed between the two MSNP-MB, mannose-decorated nanoparticles being more effective than those functionalized with amino groups. These results suggest that targeted delivery of photodynamic drugs may be a promising strategy for the treatment of localized bacterial infections.

## 3. Experimental Section 

### 3.1. Chemicals

Tetraethyl orthosilicate (TEOS), 3-(triethoxysilyl)propyl isocyanate (Si-NCO), *d-*mannosamine hydrochloride, cetyltrimethylammonium chloride solution (25 wt % in H_2_O), methylene blue (MB), Dubelcco’s phosphate buffered saline (PBS), and absolute ethanol (EtOH) were purchased from Sigma-Aldrich (St. Louis, MO, USA) and used as received. Ammonia solution (30 wt % in H_2_O) and glacial acetic acid were supplied by Panreac (Barcelona, Spain). 

### 3.2. Synthesis and Functionalization of Mesoporous Silica Nanoparticles

#### 3.2.1. Synthesis of *N*-(*d*-Mannose)-*N*'-(3-(triethoxysilyl)propyl))-urea and 3-(Triethoxysilyl)propyl isocyanate in 1:3 Molar Ratio

3-(triethoxysilyl)propyl isocyanate (212 mg, 0.81 mmol) was added to a solution of *d-*mannosamine hydrochloride (58 mg, 0.27 mmol) and Na_2_CO_3_ (32 mg, 0.30 mmol) in a mixture of 4 mL of acetonitrile and 2 mL of EtOH. The solution was stirred for 5 h at room temperature and solvent was removed under reduced pressure. Finally, the crude was diluted with 2 mL of EtOH and used without further purification.

IR (KBr disk): 3431, 2929, 1652, 1402, 1076 cm^−1^.

#### 3.2.2. Synthesis of MSNP

One hundred twenty microliters of ammonia solution (30% *w*/*w*, 1.9 mmol) were added to a solution of water (65 mL), EtOH (11.5 mL), and CTAC (7.97 mmol). The mixture was heated at 48 °C and stirred at 1000 rpm for 30 min. Then, 7.3 mL of TEOS (32.7 mmol) were added dropwise and the reaction was left stirring at 48 °C for 2 h. The crude was left aging for 24 h at room temperature. Afterwards, 20 mL of HCl (37% *w*/*w*) were added under stirring and kept at room temperature for 12 h. MSNP were obtained after centrifugation at 6000 rpm for 30 min and were put in suspension in EtOH (ultrasounds). This extraction procedure was repeated 3 times and the final nanoparticles were stored in 95 mL of EtOH at room temperature.

#### 3.2.3. Synthesis of AMSNP

Nineteen milliliters of MSNP were centrifuged for 30 min at 6000 rpm and the pellet suspended in 2 mL of EtOH under sonication. Next, 2 mL of a solution of 3-(triethoxysilyl)propyl isocyanate (212 mg, 0.81 mmol) were added dropwise under vigorous stirring (1000 rpm). The mixture was kept at 33 °C for 24 h and centrifuged at 6000 rpm for 30 min. The solvent was removed and the solid was suspended with 19 mL of water 3 times. Finally, the AMSNPs were washed 3 times with 19 mL of EtOH and stored at room temperature.

#### 3.2.4. Synthesis of MMSNP

Nineteen milliliters of MSNP were centrifuged for 30 min at 6000 rpm and the pellet suspended in 2 mL of EtOH under sonication. Next, 2 mL of the solution of *N*-(*d*-mannose)-*N*'-(3-(triethoxysilyl)propyl))-urea and 3-(triethoxysilyl)propyl isocyanate in a 1:3 molar ratio were added dropwise under vigorous stirring (1000 rpm). The mixture was kept at 33 °C for 24 h and centrifuged at 6000 rpm for 30 min. The solvent was removed and the solid was suspended with 19 mL of water 3 times. Finally, the MMSNPs were washed 3 times with 19 mL of EtOH and stored at room temperature.

#### 3.2.5. Loading of MB onto MSNP, AMSNP, or MMSNP

Two milliliters of a stock 1-mM MB solution in ethanol were added to 19 mL of each set of the nanoparticle suspensions. The mixture was stirred at 800 rpm for 48 h and the nanoparticles were isolated through repeated centrifugations. Several washes with EtOH were performed until a white supernatant was achieved. 

### 3.3. Techniques for the Characterization of Nanostructures

#### 3.3.1. Determination of the Size, ζ-Potential, and Infrared Spectra of MSNP

Size and ζ-potential of the as-synthesized mesoporous silica nanoparticles were measured using a Nano-ZS Zetasizer equipment (Malvern Instruments LtD, Worcestershire, UK). For size examination, a diluted aliquot of the nanostructures in ethanol was used. Zeta potential was determined using a diluted aliquot in milli-Q water.

Infrared spectra of the nanoparticles supported on a potassium bromide disk were recorded in a Nicolet Magna 560 FTIR spectrophotometer.

#### 3.3.2. Quantification of the MB Loading

Accurate quantification of the MB loading onto the nanostructures was achieved by measuring the concentration of unbound MB ([MB]) in the solvent phase. Specifically, the nanoparticles were separated by centrifugation and the supernatant solution was analyzed using absorption spectroscopy. The separated nanoparticles were then re-suspended in ethanol to their original concentration and the procedure was repeated until no further MB could be extracted, which required typically 6–7 cleanup cycles. The concentration of the loaded MB was calculated using Equation (2):

(2) $${\left[\text{MB}\right]}_{\text{loaded}}={\left[\text{MB}\right]}_{\text{total}}-(\sum_{i}{\left[\text{MB}\right]}_{\text{supernatant}-i})$$

where [MB]_loaded_ is the concentration of the loaded MB in the suspension, [MB]_total_ is the initial concentration of MB, and [MB]_supernatant-*i*_ is the concentration of MB in each supernatant determined by absorption spectroscopy.

Additionally, the MB-loading ratio (MB-LR) was calculated using Equation (3):

(3) $$\text{MB}-\text{LR}\left(\%\right)=\frac{{\left[\text{MB}\right]}_{\text{loaded}}}{{\left[\text{MB}\right]}_{\text{total}}}\times 100$$

### 3.4. Spectroscopic Techniques

The photophysical properties of AMSNP-MB and MMSNP-MB were measured in absolute ethanol (EtOH) and compared to MB under the same conditions. In order to remove the light scattering contribution of MSNPs, their absorption spectra were recorded using “white” (no MB-loaded) MSNPs as reference. Absorption spectra were recorded with a Varian Cary 6000i spectrophotometer. Fluorescence spectra were measured with a Fluoromax-4 spectrofluorometer. 

Time-resolved fluorescence experiments were carried out using a customized PicoQuant Fluotime 200 fluorescence lifetime system. The fluorescence was excited at 596 nm by means of a pulsed LED working at 10 MHz repetition rate, and was observed at the emission maxima keeping the counting frequency below 1%. Fluorescence decays were analyzed using the PicoQuant FluoFit v4.6.5 data analysis software.

For time-resolved phosphorescence detection, a diode-pumped Nd:YAG laser (FTSS355-Q, Crystal Laser, Berlin, Germany) was used for excitation at 532 nm working at 1 kHz repetition rate (1.2 μJ per pulse, 1 ns pulse-width). A 1064-nm rugate notch filter (Edmund Optics, U.K.) was placed at the exit port of the laser to remove any residual component of its fundamental emission in the NIR region. The luminescence exiting from the side of the sample was filtered by one long-pass filter of 1000 nm and narrow bandpass filters at either 1270 or 1110 nm to remove any scattered laser radiation and isolate the NIR emission. A TE-cooled Hamamatsu NIR sensitive photomultiplier tube assembly (H9170-45, Hamamatsu) was used as detector. Photon counting was achieved with a multichannel scaler (PicoQuant’s Nanoharp 250).

In order to vary the concentration of oxygen in the solution, a stream of oxygen 5.0 (Carburos Metálicos) was flowed above the MSNP suspension under gentle stirring for ca. 30 min.

### 3.5. Microbial Techniques

#### 3.5.1. Microbial Strains and Growth Conditions

*Escherichia coli* CECT 101 and *Pseudomonas aeruginosa* CECT 116 were obtained from the Spanish Type Culture Collection (CECT). Bacterial cells were grown overnight in sterile tryptic soy broth at 37 °C. Stock inoculum suspensions were prepared in PBS and adjusted to an optical density at 600 nm of 0.4 for *E. coli* and 0.6 and *Pseudomonas aeruginosa* (equivalent to ca. 10^8^ colony-forming units).

#### 3.5.2. Photodynamic Inactivation Procedure

Cell suspensions in PBS were incubated with the PS (free MB, AMSNP-MB, or MMSNP-MB) for 30 min in the dark at room temperature. Then, 0.3 mL of the suspensions was placed in 96-well plates. Excluding dark controls, the plates were illuminated from the top by a LED-based lamp emitting red light (625 ± 20 nm) for 15 or 30 min (fluences of 16 and 32 J/cm^2^ respectively), then serially diluted 10 times, seeded on tryptic soy agar, and incubated for 24 h at 37 °C. Colony-forming units (CFU) were counted to calculate the survival fractions.

## 4. Conclusions 

MB adsorbed on amino- or mannose-functionalized mesoporous silica nanoparticles is capable of efficiently inactivating *E. coli* and *P. aeruginosa* bacteria upon exposure to red light. The photodynamic activity is similar to that of free MB but, in the case of *E. coli*, the dark toxicity is improved. In the case of *P. aeruginosa*, mannose is a better targeting agent than free amino groups on the surface of the nanoparticle. Time-resolved spectroscopic studies reveal the existence of two different populations of MB in the nanoparticles, the major one being adsorbed on the mesopores’ walls and the minor on the external surface of the nanoparticle. Strong quenching of singlet oxygen in the pores of the nanoparticle is observed. 
